# P-1567. Prevalence of the acrB Gene in Class 1 Integrons Among Carbapenem-Resistant Enterobacterales in Southern Taiwan

**DOI:** 10.1093/ofid/ofaf695.1747

**Published:** 2026-01-11

**Authors:** Susan Shin-Jung Lee, Hui-Ling Hsia, Yi-Ting Lee, Hsi-Hsun Lin

**Affiliations:** Kaohsiung Veterans General Hospital, Kaohsiung, Kaohsiung, Taiwan (Republic of China); Kaohsiung Veterans General Hospital, Kaohsiung, Kaohsiung, Taiwan (Republic of China); Kaohsiung Veterans General Hospital, Kaohsiung, Kaohsiung, Taiwan (Republic of China); Kaohsiung Veterans General Hospital, Kaohsiung, Kaohsiung, Taiwan (Republic of China)

## Abstract

**Background:**

Acriflavine resistance protein B (*acrB*) serves as the drug specificity and energy transduction component of the AcrAB-TolC multidrug efflux system. This study aimed to investigate the prevalence of the *acrB* gene within class 1 integrons among carbapenem-resistant *Enterobacterales* (CRE) clinical isolates in Southern Taiwan.

**Table 1.**
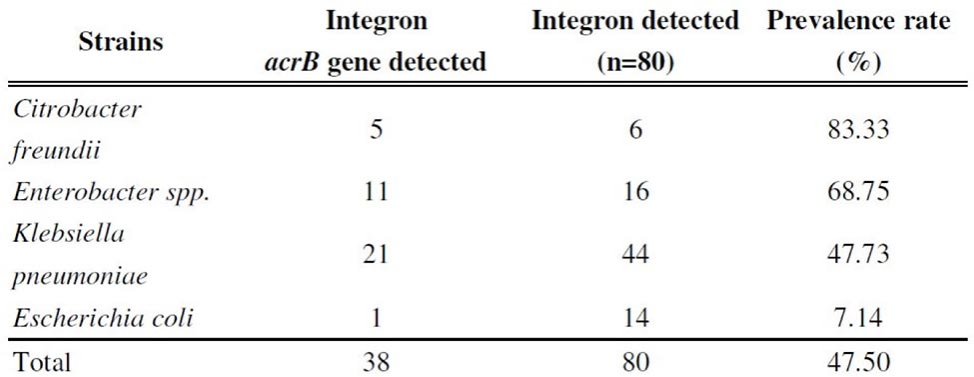
Class 1 Integron Cassettes Carrying acrB genes detected by PCR in clinical isolates of CP-producing CRE in Southern Taiwan (N=80)

**Table 2.**
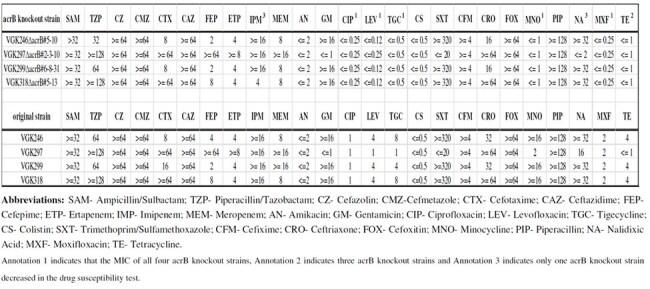
Comparison of Minimum Inhibitory Concentrations (MICs, µg/mL) Between acrB Gene Knockout Strains and Parental Strains in Antimicrobial Susceptibility Testing

**Methods:**

A total of 101 CRE isolates were collected from 2020 to 2023. Genomic DNA was extracted, followed by PCR detection of class 1 integron genes. These integron gene templates were subsequently used for PCR amplification and sequencing of the *acrB* and carbapenemase genes. *acrB* knockout strains were constructed, and the minimum inhibitory concentrations (MICs) of selected antibiotics were determined using the Vitek 2 system.

**Results:**

Class 1 integrons were detected in 79.2% (80/101) of CRE isolates. Among these 47.5% (38/80) harbored the *acrB* gene. The highest prevalence of integron-associated *acrB* was found in *Citrobacter freundii* (83.3%, 5/6), followed by *Enterobacter* spp. (68.8%, 11/16), *Klebsiella pneumoniae* (47.7%, 21/44), and *Escherichia coli* (7.1%, 1/14). MICs for ciprofloxacin, levofloxacin, tigecycline, minocycline, moxifloxacin, and tetracycline were reduced by 2- to 16-fold in *acrB* knockout strains compared to their corresponding parental strains.However, no changes were observed in carbapenem susceptibility.

**Conclusion:**

This study reveals a high prevalence of the *acrB* gene within class 1 integrons among CRE isolates in Southern Taiwan. The observed reduction in MICs in *acrB* knockout mutants supports the role of AcrB in multidrug resistance and emphasizes the need for further research into its functional role and evolutionary significance.

**Disclosures:**

All Authors: No reported disclosures

